# Hydrothermal Conversion of Annatto Seed Waste (*Bixa orellana*) into Functional Hydrochar: Synthesis, Characterization, and Adsorption Mechanism of Tetracycline

**DOI:** 10.3390/molecules31071224

**Published:** 2026-04-07

**Authors:** Diana Guaya, Linda Jadán, José Luis Cortina

**Affiliations:** 1 Department of Chemistry, Universidad Técnica Particular de Loja, Loja 110107, Ecuador; 2 Escuela de Ingeniería Química, Universidad Técnica Particular de Loja, Loja 110107, Ecuador; 3 Department of Chemical Engineering, Polytechnic University of Catalonia–BarcelonaTech (UPC), 08019 Barcelona, Spain; jose.luis.cortina@upc.edu; 4 Barcelona Research Center in Multiscale Science and Engineering (CCEM), Polytechnic University of Catalonia–BarcelonaTech (UPC), Av. Eduard Maristany, 16, 08019 Barcelona, Spain

**Keywords:** hydrothermal carbonization, annatto, biomass valorization, adsorption kinetics, tetracycline

## Abstract

Agroindustrial residues represent an abundant and underutilized source of carbon-rich materials for environmental remediation. In this study, annatto processing waste (*Bixa orellana*), a largely unexplored lignocellulosic by-product generated during pigment extraction, was converted into hydrochar via hydrothermal carbonization at 200 °C for 3 h. The resulting hydrochar (HC-AW) exhibited a predominantly amorphous carbon structure with retained oxygen-containing surface functionalities, and a solid yield of 44%, indicating efficient biomass conversion under subcritical conditions. Adsorption performance toward tetracycline was evaluated through pH-dependent experiments, kinetic modeling, equilibrium isotherms, and thermodynamic analysis. Maximum adsorption occurred under near-neutral conditions (pH ≈ 7), consistent with the interplay between tetracycline speciation and the hydrochar surface charge (pH_PZC_ ≈ 6.3), highlighting its potential applicability under realistic water treatment conditions without pH adjustment. Kinetic data were well described by the pseudo-second-order model, while equilibrium results were best fitted by the Langmuir model, with a maximum adsorption capacity of 14.94 mg g^−1^ at 30 °C. Thermodynamic analysis indicated a spontaneous and slightly endothermic adsorption process. Overall, the results highlight the potential of annatto-derived hydrochar as a low-cost adsorbent and provide insight into the relationship between surface properties and adsorption behavior governing antibiotic removal from aqueous systems.

## 1. Introduction

Agroindustrial chains generate substantial quantities of lignocellulosic by-products whose inadequate management leads to environmental externalities and the underutilization of potentially valuable carbon-rich resources for chemical and materials applications [1,2]. Annatto (*Bixa orellana*), widely processed as a natural source of pigments for food, cosmetic, and pharmaceutical applications, generates a solid residue after extraction that remains enriched in organic macromolecules and carbonaceous components [3]. Recent reports estimate global annatto production to be on the order of 14,500–17,000 tons annually, with Latin America accounting for the largest share. Since pigment extraction is primarily associated with the aril fraction, which represents only 5–10% of the seed mass, industrial processing generates a lignocellulosic residue that remains largely underexplored [4]. Beyond conventional disposal, similar agroindustrial residues have been investigated as feedstocks for value-added applications within circular-economy frameworks, including the production of bioenergy carriers, soil amendments, bioactive compounds, and functional carbonaceous materials [5]. However, for annatto-processing residues in particular, there remains limited information on conversion pathways capable of generating adsorption-relevant surface properties under mild and scalable processing conditions [4,6].

Hydrothermal carbonization (HTC) has emerged as an attractive wet-chemistry route for converting moist biomass into hydrochar under subcritical water conditions, typically at lower temperatures (150–280 °C) than conventional pyrolysis and without the requirement for extensive pre-drying [7]. In contrast to biochars produced by dry thermochemical processes, hydrochars commonly display a higher abundance of oxygen-containing surface functionalities, such as hydroxyl, carbonyl, and carboxyl groups, which can impart distinct polarity, acid–base behavior, and pH-dependent surface charge characteristics [8]. These attributes are particularly relevant to adsorption processes, where the nature and distribution of surface functional groups influence electrostatic interactions, hydrogen bonding, and other specific affinity mechanisms [9]. From a process perspective, annatto residues can, in principle, be transformed through several thermochemical and hydrothermal routes (e.g., pyrolysis, gasification, torrefaction, and HTC), with yields and material properties governed by feedstock composition and synthesis parameters. Importantly, HTC provides a complementary platform to thermally driven carbonization strategies previously reported for annatto-derived carbon materials, enabling a consistent comparison of wet versus dry conversion routes in terms of surface chemistry and adsorption-relevant functionality. In addition, it offers a lower-energy pathway for biomass conversion, particularly suited to decentralized and resource-limited contexts, where agroindustrial residues such as annatto waste represent a sustainable and underutilized feedstock [10].

The presence of pharmaceuticals in aquatic environments has become a critical issue, and antibiotics are of particular concern due to their extensive use in human therapy, veterinary medicine, and agriculture, representing approximately 10–12% of the global antimicrobial market [11]. Multiple pathways contribute to their release into water bodies, including the excretion of parent compounds and metabolites. Notably, 70–90% of administered tetracyclines are excreted in an unaltered form [12]. Additional sources include discharges from healthcare and livestock activities, as well as the improper disposal of medications [13]. These continuous inputs are especially problematic because antibiotics can exert selective pressure on microbial communities, thereby promoting the emergence and dissemination of antimicrobial resistance (AMR), often described in public discourse as “superbugs” [14]. In addition, conventional wastewater treatment infrastructures are frequently not designed as dedicated barriers for micropollutant antibiotics, resulting in incomplete removal and persistence in receiving waters [15]. Consequently, the development of complementary treatment strategies capable of addressing antibiotics under realistic water-chemistry conditions remains a priority [16].

A variety of technologies have been investigated for the removal of antibiotics from aqueous systems, including biological treatments, advanced oxidation processes (AOPs), membrane-based separations, and adsorption [17]. Conventional biological treatments are often limited by the recalcitrant nature of many antibiotics and their incomplete biodegradation [18]. Advanced oxidation processes, such as ozonation, photocatalysis, and Fenton-based systems, can achieve high degradation efficiencies; however, they are typically associated with high energy demand, chemical consumption, and the potential formation of toxic transformation products [19,20,21,22]. Membrane technologies, including nanofiltration and reverse osmosis, offer effective physical separation but are constrained by membrane fouling, operational costs, and the need for concentrate management [23]. In this context, adsorption has emerged as a versatile and cost-effective alternative due to its operational simplicity, scalability, and compatibility with tertiary treatment systems [24]. Biomass-derived carbonaceous materials, particularly hydrochars, have attracted increasing attention as adsorbents because of their tunable surface chemistry and potential for sustainable production [25]. Importantly, because hydrochar can be produced from locally available agroindustrial wastes using comparatively mild processing conditions, it represents a low-cost and scalable platform for developing functional adsorbents for antibiotic-contaminated waters [26].

Tetracycline is a particularly relevant model molecule because its adsorption is strongly governed by acid–base speciation. It exhibits pK_a_ values of approximately 3.3, 7.7, and 9.7, which define its predominant species as cationic at acidic pH, zwitterionic under near-neutral conditions, and anionic at alkaline pH [27]. As a result, adsorption depends not only on the textural properties of the adsorbent, but also on the relationship between solution pH, tetracycline speciation, and the pH-dependent surface charge of the material. This interplay governs the balance between electrostatic attraction and repulsion, as well as other interaction mechanisms [28]. Consequently, the adsorption behavior of tetracycline is highly sensitive to pH conditions, making the understanding of speciation–surface charge relationships essential for interpreting adsorption performance.

The novelty of the present work lies in two complementary aspects. First, it explores annatto (*Bixa orellana*) agroindustrial residue as a precursor for hydrochar production via hydrothermal carbonization, a valorization route that remains scarcely documented and, to the best of our knowledge, has not been specifically established for tetracycline adsorption. Second, beyond reporting adsorption performance, this study aims to elucidate the structure–surface chemistry–adsorption relationships governing tetracycline uptake by correlating hydrothermal conversion conditions, surface functionality, pH_PZC_ behavior, pH-dependent tetracycline speciation, and adsorption response. Accordingly, the objectives of this study are: (i) to synthesize annatto-residue-derived hydrochar under controlled hydrothermal conditions; (ii) to characterize its physicochemical and surface properties using complementary techniques, with emphasis on adsorption-relevant descriptors; and (iii) to evaluate tetracycline adsorption through pH-dependent experiments, kinetic and equilibrium analyses, and mechanistic interpretation. In this way, this study provides not only a new biomass-derived hydrochar platform, but also mechanistic insight into how HTC-derived surface features from an underexplored industrial residue influence antibiotic uptake, thereby offering a reproducible basis for further material optimization and for the development of sustainable, cost-effective adsorbents for pharmaceutical mitigation in water systems.

## 2. Results and Discussions

### 2.1. Hydrochar Synthesis and Yield

Hydrothermal carbonization of annatto (*Bixa orellana*) agroindustrial residue produced a carbonaceous solid with a yield of 44% (dry basis). This value reflects effective biomass conversion under subcritical conditions (200 °C, 3 h) and suggests a balance between solid-phase preservation and thermochemical transformation. To the best of our knowledge, no previous yield data have been reported for annatto-derived residues; therefore, this result provides a novel reference for this biomass and establishes a basis for comparison with other lignocellulosic feedstocks. To the best of our knowledge, no previous yield data have been reported for annatto-derived residues. Consequently, this result provides a novel reference for this biomass and establishes a basis for comparison with other lignocellulosic feedstocks.

**Figure 1:**
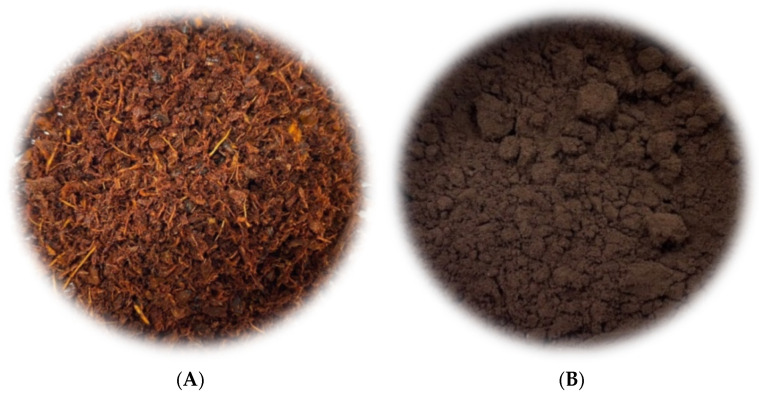
Visual appearance of annatto agroindustrial residue before hydrothermal treatment AW (**A**) and the corresponding hydrochar obtained after hydrothermal carbonization HC-AW (**B**).

The hydrothermal treatment was accompanied by a clear visual transformation of the material. The raw annatto (AW) residue, originally reddish–orange due to the residual pigments (Figure 1A), was converted into a dark brown to nearly black solid (Figure 1B), representing a qualitative indication of material transformation under hydrothermal conditions.

The yield obtained via hydrothermal carbonization is notably higher than that typically associated with dry thermochemical treatments of annatto waste, such as pyrolysis, where higher operating temperatures promote extensive devolatilization and carbon loss in the form of gaseous products. For instance, annatto-derived biochar produced under inert conditions exhibited a yield of approximately 31%, reflecting the greater severity of carbon degradation at elevated temperatures [6]. In contrast, the aqueous environment characteristic of hydrothermal processing moderates thermal decomposition pathways and promotes secondary polymerization and recondensation reactions, favoring carbon retention in the solid phase [29,30].

A more detailed comparison with lignocellulosic residues processed under identical conditions (200 °C, 3 h) provides further insight into the significance of the 44% yield obtained in this study. Reported hydrochar yields for eucalyptus, giant bamboo, and coffee wood are 64.2%, 59.3%, and 65.5%, respectively, while a lower value of approximately 54.1% has been observed for coffee parchment under the same conditions [31]. These differences highlight the strong influence of biomass composition, particularly the relative content of hemicellulose, cellulose, and lignin, on hydrothermal degradation behavior. Labile fractions such as hemicellulose degrade significantly at 180–200 °C, contributing to mass loss, whereas more recalcitrant components such as lignin favor solid-phase retention. Therefore, the observed yield reflects the combined influence of process conditions and intrinsic compositional characteristics, which control the balance between decomposition and recondensation pathways. From a process perspective, these results confirm that temperature is the dominant parameter controlling hydrochar yield, while residence time (3 h in all cases) ensures sufficient progression of hydrothermal reactions without being the primary driver of mass loss. The observed variations among feedstocks therefore arise mainly from intrinsic compositional differences rather than operational conditions [32].

Overall, the observed yield reflects a balance between carbon retention and chemical transformation. While lower yields are typically associated with more extensive carbonization, the moderate yield obtained in this study is consistent with hydrochars that retain oxygen-containing surface functionalities relevant for adsorption processes. These results highlight the suitability of hydrothermal carbonization as an effective and scalable route for the valorization of annatto agroindustrial residue.

### 2.2. Physicochemical Characterization of Annatto-Derived Hydrochar

The chemical composition of annatto agroindustrial waste (AW) and the corresponding annatto-derived hydrochar (HC-AW), determined by X-ray fluorescence (XRF), is summarized in Table 1. The values correspond to relative oxide percentages within the inorganic fraction and allow a comparative assessment of mineral composition. Overall, hydrothermal carbonization preserves the inorganic elemental fingerprint of the precursor while promoting a redistribution of specific mineral components.

Magnesium oxide (MgO) was identified as a major constituent in both materials, showing a relative enrichment in HC-AW, consistent with mineral concentration following partial removal of organic matter [31]. Silicon dioxide (SiO_2_) exhibited a slight increase, indicating stability under hydrothermal conditions, as similarly reported for other biomass-derived hydrochars (e.g., cow-dung digestate) [33]. In contrast, phosphorus pentoxide (P_2_O_5_) decreased after treatment, likely due to the decomposition of organic phosphorous species and leaching into the process water [31]. Alkali and alkaline earth elements, such as K_2_O and CaO, were also detected; potassium showed a reduction due to its mobility in aqueous media, while calcium remained relatively stable in the solid matrix [34].

XRF analysis is limited in detecting light elements, such as carbon, hydrogen, and oxygen, which constitute the major fraction of hydrochar materials; therefore, energy-dispersive X-ray spectroscopy (EDS) was used as a complementary technique (Figures S1 and S2, Supplementary Material). The EDS results confirmed the predominance of carbon and oxygen in both AW and HC-AW, supporting the carbonaceous nature of the material.

The combined XRF-EDS analysis enables differentiation between the inorganic mineral fraction and the carbon-rich matrix, while also indicating that trace elements such as iron, manganese, chromium, strontium, and lead remain at low levels without enrichment after hydrothermal treatment. Overall, these results indicate that hydrothermal carbonization preserves the mineral signature of the precursor while producing a carbon-rich material, consistent with typical hydrothermal conversion processes.

The presence of inorganic components such as SiO_2_, Al_2_O_3_, CaO, and Fe_2_O_3_ may influence the functional properties of the hydrochar, including surface charge and ion-exchange capacity. Elements such as Ca and Fe are noteworthy, as similar species have been reported to enhance the performance of carbonaceous materials in adsorption applications. In this context, the incorporation or modification of hydrochars with such metal species has been widely explored to improve adsorption capacity, highlighting the potential relevance of these inorganic constituents in the present material.

To contextualize these results, a comparison with annatto seed-derived biochar previously reported [6] is informative. In that study, pyrolyzed biochar at 700 °C (ABC-N700) exhibited higher relative contents of MgO, Al_2_O_3_, SiO_2_, P_2_O_5_, S, K_2_O, CaO, and Fe_2_O_3_, with minor contributions from other elements such as Mn and Zn, reflecting the concentration of inorganic matter under high-temperature conditions.

In contrast, the HC-AW produced in this study shows comparatively lower mineral oxide content (e.g., MgO and SiO_2_), which can be attributed to the milder hydrothermal conditions (200 °C) that favor partial solubilization and redistribution of inorganic species in the aqueous phase [33].

Consequently, HC-AW retains a composition closer to the native mineral distribution of the precursor, with limited ash enrichment compared to pyrolyzed biochar [35]. This difference highlights the influence of the synthesis route on material composition, with hydrothermal carbonization yielding a more organic-rich and less mineral-concentrated carbonaceous material [36].

**Figure 2:**
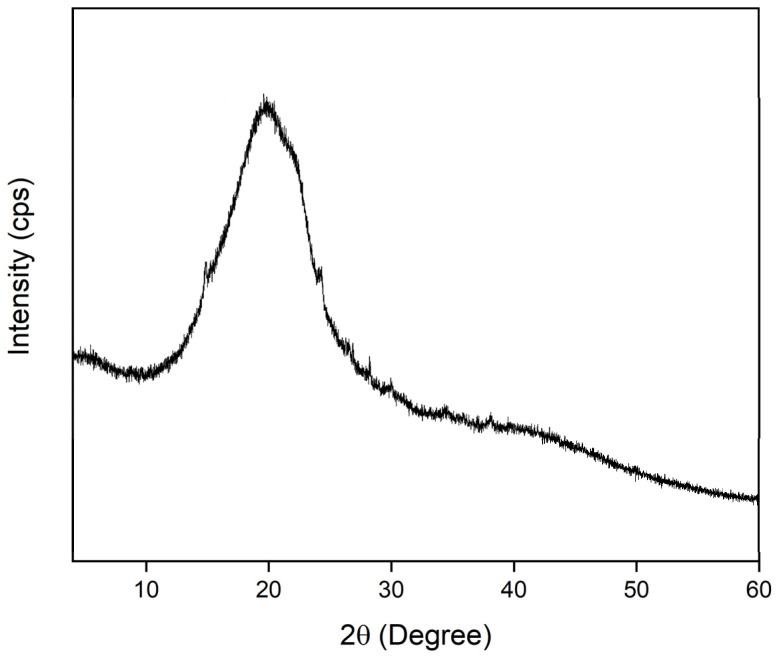
X-ray diffraction (XRD) pattern of annatto-residue-derived hydrochar (HC-AW).

The X-ray diffraction (XRD) pattern of the annatto-derived hydrochar (HC-AW) is shown in Figure 2. The diffractogram is dominated by broad and diffuse features rather than sharp, well-defined reflections, indicating the predominantly amorphous nature of the material. This structural behavior is typical of hydrochars obtained from lignocellulosic biomass under mild hydrothermal conditions, where the processing temperature is insufficient to induce significant structural ordering [37].

A broad diffraction band centered in the 2θ region of approximately 20–25° is observed in the XRD pattern of HC-AW, a feature commonly reported in hydrochars derived from lignocellulosic biomass and associated with predominantly amorphous carbon structures lacking long-range crystallographic order [38]. This profile reflects disordered carbonaceous matrices composed of small aromatic domains with limited structural coherence. In contrast to the sharper reflections of graphitic carbon formed at higher temperatures, this broad band indicates short-range structural organization developed under subcritical hydrothermal conditions, where condensation occurs without sufficient thermal severity for graphitization [39]. These structural features indicate that HC-AW consists of a predominantly disordered carbon matrix, consistent with the formation of hydrochar from annatto waste.

A direct comparison between the XRD pattern of HC-AW and the previously reported annatto-derived biochar highlights the structural divergence induced by the synthesis route. While the pyrolyzed biochars obtained at 700 °C exhibited discernible crystalline reflections superimposed on broad carbon halos, indicative of increased structural ordering and concentration of mineral phases [6], the hydrochar synthesized at 200 °C displays exclusively broad, diffuse features characteristic of an amorphous carbon framework.

These differences arise from the distinct processing conditions: high-temperature pyrolysis promotes carbon condensation and short-range structural ordering, along with mineral concentration due to devolatilization of organic matter, leading to more defined diffraction features [40]. In contrast, hydrothermal carbonization under subcritical conditions favors dehydration and polymerization reactions without sufficient energy for graphitic organization, resulting in a predominantly disordered carbon structure with no evidence of extended crystalline domains [38].

**Figure 3:**
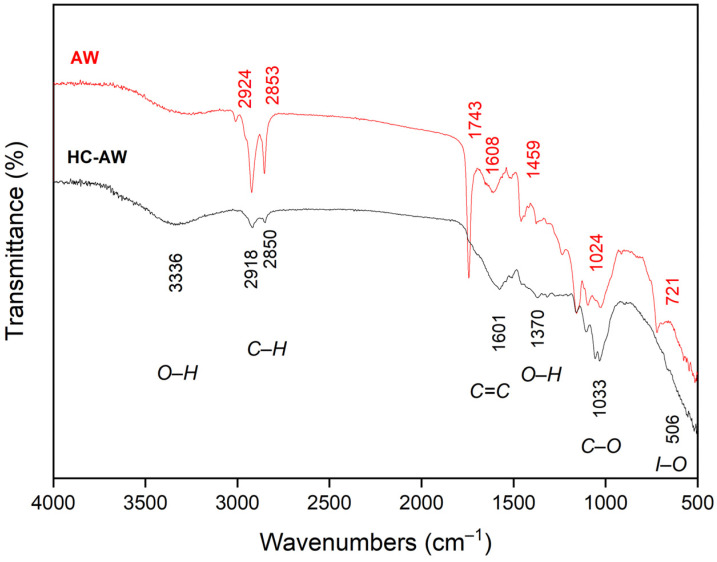
FTIR spectra of annatto-waste (AW) and annatto-derived hydrochar (HC-AW). The band assignment I-O corresponds to inorganic–O functional groups.

The FTIR spectra of annatto agroindustrial waste (AW) and annatto-derived hydrochar (HC-AW) are presented in Figure 3, evidencing the structural transformation induced by hydrothermal carbonization.

The broad band in the 3350–3330 cm^−1^ region, attributed to O–H stretching of hydroxyl groups from cellulose, hemicellulose, lignin, and adsorbed water, is observed in both materials [41], although with reduced intensity and altered profile in HC-AW, indicating partial dehydration and reorganization of hydrogen-bonded structures [42].

In the aliphatic region (2924 and 2853 cm^−1^), AW shows characteristic C–H stretching vibrations of–CH_2_ and –CH_3_ groups [43], which decrease in intensity and slightly shift after hydrothermal treatment (2918 and 2850 cm^−1^). This behavior reflects partial degradation of aliphatic chains and structural rearrangement within the carbon matrix [44].

The carbonyl region shows significant changes, with the band at 1743 cm^−1^ (ester and carboxylic groups) markedly reduced in HC-AW, suggesting decarboxylation of oxygenated structures. Concurrently, the band near 1608 cm^−1^ in AW shifts to 1601 cm^−1^ in HC-AW and becomes more defined, indicating the relative enrichment of aromatic and conjugated domains [45].

In the fingerprint region (1024 cm^−1^), AW exhibits a C–O stretching band associated with polysaccharides [46], which decreases in intensity and shifts slightly to 1033 cm^−1^, evidencing partial degradation of carbohydrate structures while confirming the persistence of oxygen-containing functionalities such as ethers and phenols [47].

Additional attenuation of bands in the range of 1480–1370 cm^−1^ region and at 721 cm^−1^, further supports structural reorganization of aliphatic and aromatic groups (C–H bending). The low-wavenumber band around 506 cm^−1^ in HC-AW is attributed to inorganic–oxygen vibrations (e.g., phosphate (P–O), silicate (Si–O) and metal–oxygen (M–O)) [47], consistent with the mineral phases identified by XRF and EDS analyses. Overall, the FTIR results indicate that hydrothermal carbonization at 200 °C promotes the transformation of labile aliphatic and oxygenated groups while retaining a significant fraction of oxygen-containing functionalities within a reorganized carbon matrix, consistent with the typical chemical characteristics of hydrochars produced under subcritical conditions.

A direct comparison between the FTIR spectrum of HC-AW (Figure 3) and that previously reported for natural annatto biochar (ABC-N700) highlights differences in functional group distribution associated with the distinct carbonization pathways. The biochar produced at 700 °C shows attenuation of aliphatic C–H bands, predominance of aromatic C=C vibrations [6], and reduced oxygenated functionalities consistent with advanced thermal decomposition and deoxygenation [48].

In contrast, HC-AW retains O–H, C–O, and C=O vibrations, indicating the preservation of oxygen-containing functional groups under hydrothermal conditions at 200 °C. These differences reflect the influence of processing conditions, where pyrolysis promotes deoxygenation and structural condensation, while hydrothermal carbonization preserves a more oxygen-rich functional framework [6].

**Figure 4:**
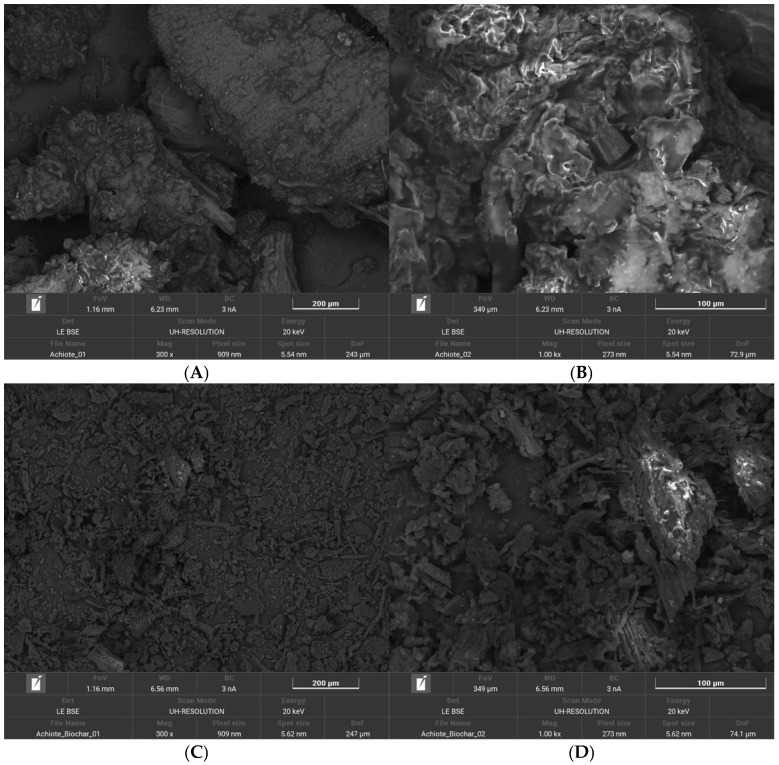
SEM micrographs of annatto agroindustrial waste (AW) and annatto-derived hydrochar (HC-AW) at different magnifications: (**A**) AW at 50 µm; (**B**) AW at 5 µm; (**C**) HC-AW at 50 µm; (**D**) HC-AW at 5 µm.

The surface morphology of annatto agroindustrial waste (AW) and the corresponding annatto-derived hydrochar (HC-AW) was examined by scanning electron microscopy (SEM) at two magnifications (50 µm and 5 µm), as shown in Figure 4. Limited morphological information is available for annatto waste generated after industrial pigment extraction, as most studies have focused on the seed structure itself. The AW material exhibits heterogeneous lignocellulosic features, including elongated structures and partially preserved plant tissue organization, with relatively compact surfaces and limited visible macroporosity. At higher magnification (5 µm; Figure 4B), the surface appears smooth, with layered cell-wall fragments and intact fibrillar domains, indicating preservation of the original biomass structure prior to hydrothermal treatment [49].

After hydrothermal carbonization, HC-AW shows a clear morphological transformation. At low magnification 50 µm (Figure 4C), the fibrous structure is disrupted, leading to fragmented and irregular aggregates with loss of structural continuity. At a higher magnification of 5 µm (Figure 4D), the surface exhibits increased roughness, with fissures and microscale cavities not observed in the raw material. These changes are consistent with the structural reorganization of lignocellulosic biomass under hydrothermal conditions, resulting in heterogeneous surfaces with limited pore development [32]. Although microscale cavities are present, HC-AW does not exhibit well-defined and interconnected pore structures, in contrast to biochars produced under high-temperature pyrolysis conditions [6]. Overall, the observed morphological features confirm the structural transformation of the precursor biomass into a hydrochar with a heterogeneous and disordered surface, consistent with hydrothermal carbonization pathways and distinct from materials obtained via high-temperature pyrolysis.

### 2.3. Adsorption of Tetracycline

#### 2.3.1. Effect of pH on Adsorption

**Figure 5:**
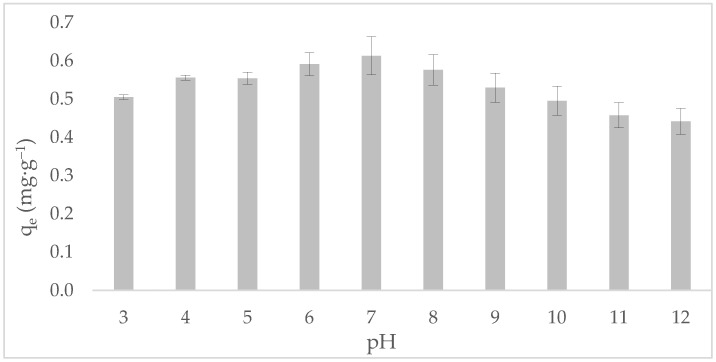
Influence of solution pH on tetracycline adsorption capacity of annatto-derived hydrochar (HC-AW).

The influence of solution pH on tetracycline (TC) adsorption onto annatto-derived hydrochar (HC-AW) is shown in Figure 5. The adsorption behavior exhibits a pronounced dependence on pH, which can be systematically interpreted by considering (i) the acid–base speciation of tetracycline and (ii) the surface charge behavior of the hydrochar as defined by its point of zero charge (pH_PZC_).

The pH_PZC_ of HC-AW was experimentally determined as 6.3 ± 0.5, indicating that the hydrochar surface is positively charged at pH < pH_PZC_ and negatively charged at pH > pH_PZC_. Tetracycline is an amphoteric molecule characterized by three dissociation constants (pK_a1_ ≈ 3.3, pK_a2_ ≈ 7.7, and pK_a3_ ≈ 9.7), which define its speciation profile in aqueous media [50]. At pH < 3.3, TC predominantly exists in its cationic form (TC^+^). Under acidic conditions (pH < 3), the adsorption capacity is limited due to electrostatic repulsion between positively charged tetracycline and the hydrochar surface. The residual uptake observed experimentally can therefore be attributed to non-electrostatic contributions. As pH increases to near-neutral values (3.3 < pH < 7.7), adsorption reaches a maximum. In this region, tetracycline predominantly exists in zwitterionic form (TC^±^), while the hydrochar surface approaches charge neutrality near its p_HPZC_, minimizing electrostatic barriers and favoring adsorption. Above pH 7.7, tetracycline becomes negatively charged as monoanionic (TC^−^) and subsequently dianionic (TC^2−^) forms [51], and the hydrochar surface also acquires a negative charge, leading to electrostatic repulsion and a decrease in adsorption capacity. These results highlight the critical role of pHPZC in regulating adsorption, as it defines the surface charge conditions under which electrostatic interactions are minimized or enhanced. The alignment between the adsorption profile, tetracycline speciation, and the pH_PZC_ of HC-AW indicates that adsorption behavior can be rationally interpreted based on acid–base and surface charge considerations.

In a comparative study of hydrochar-based adsorbents for tetracycline removal, pH_PZC_ values around neutral pH ~6 have been reported for hydrochars [52], while modifications can shift this value depending on the density and nature of oxygen-containing surface groups [53]. Similarly, biochars derived from anaerobically digested sludge also show pH_PZC_ values close to 6, indicating that biomass-derived carbon materials commonly exhibit near-neutral surface charge behavior [54], although lower values may be observed in chemically modified systems due to the introduction of acidic functional groups [55].

This near-neutral pH_PZC_ is particularly favorable for tetracycline adsorption, as it enables efficient adsorption under circumneutral conditions where the zwitterionic species predominates, thereby facilitating intermolecular interaction.

#### 2.3.2. Adsorption Kinetics

**Figure 6:**
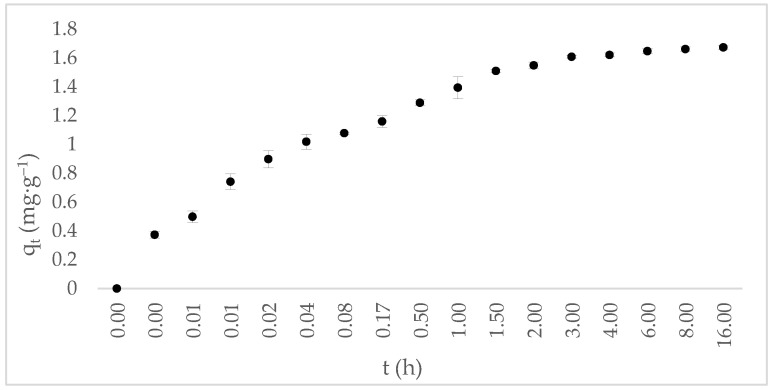
Effect of contact time on tetracycline adsorption onto annatto-derived hydrochar.

The effect of contact time on tetracycline (TC) adsorption onto annatto-derived hydrochar (HC-AW) is presented in Figure 6. The kinetic profile shows a rapid initial uptake during the early stages of contact, followed by a gradual deceleration as the system approaches equilibrium. The rapid initial uptake indicates strong interaction between TC and the hydrochar surface, while the plateau after 3 h reflects the establishment of adsorption equilibrium. Approximately 90% of the adsorption capacity is achieved within the first 2 h, indicating rapid occupation of readily available surface sites. Equilibrium is reached after approximately 3 h, with no significant increase in adsorption capacity beyond this point. The subsequent deceleration in adsorption rate suggests progressive saturation of high-energy sites and a transition toward a diffusion-influenced regime, typical of adsorption systems where initial uptake is controlled by surface interactions and later stages by mass transfer limitations.

To interpret the adsorption kinetics, the experimental data were fitted using the pseudo-first order (PFO, Equation (1)) and pseudo-second order (PSO, Equation (2)).

(1) $$\ln\left(q_{e}-q_{t}\right)=\ln\left(q_{e}\right)-k_{1}t$$

(2) $$\frac{t}{q_{t}}=\frac{1}{k_{2}q_{e}^{2}}+\frac{t}{q_{e}}$$

where q_e_ and q_t_ represent the equilibrium and time-dependent adsorption capacities (mg·g^−1^), respectively, and k_1_ (h^−1^) and k_2_ (g·mg^−1^·h^−1^) are the rate constants of the pseudo-first-order and pseudo-second-order models. Diffusion models were applied to evaluate mass-transfer contributions: intraparticle diffusion describes transport within the adsorbent (Equation (3)), film diffusion accounts for external boundary layer resistance (Equation (4)), and particle diffusion represents internal diffusion within the solid matrix (Equation (5)) [56]. The corresponding kinetic parameters are summarized in Table 2.

(3) qt=ktt12+A

(4) $$-\ln\left(1-\left(\frac{q_{t}}{q_{e}}\right)\right)=\frac{d_{f}c_{s}}{hrc_{z}}t$$

(5) $$-\ln\left(1-{\left(\frac{q_{t}}{q_{e}}\right)}^{2}\right)=\frac{2\pi^{2}d_{p}}{r^{2}}t$$

where k_t_ (mg·g^−1^·h^−1/2^) is the intraparticle diffusion rate constant, and A (mg·g^−1^) is related to boundary layer thickness, c_s_ (mg·L^−1^) and c_z_ (mg·kg^−1^) are to TC concentrations in solution and adsorbent, respectively, r is the particle radius (below 200 mesh ≈ 3.7 × 10^−5^ m), t is contact time (h) and h is film thickness (1 × 10^−5^ m for a poorly stirred solution).

The pseudo-second-order model provides the best fit to the experimental data (R^2^ = 1.00), outperforming the pseudo-first-order model (R^2^ = 0.70), and yields an equilibrium adsorption capacity (q_e_ = 1.68 mg·g^−1^) consistent with the experimental trend, whereas the PFO model underestimates adsorption capacity (q_e_ = 0.55 mg·g^−1^). This agreement indicates that the adsorption process is strongly influenced by the availability of surface sites and interactions between tetracycline and the hydrochar surface [57]; however, no definitive mechanistic assignment can be made based on kinetic modeling alone, as contributions from multiple processes may coexist.

The intraparticle diffusion model shows three linear regions (k_t1_, k_t2_, k_t3_ with R^2^ ≥ 0.97), suggesting a multi-step process involving: (i) rapid surface uptake, (ii) followed by diffusion into internal domains, and (iii) final equilibrium stabilization [58]. Intraparticle diffusion is not the sole rate-controlling step, as film diffusion (R^2^ = 0.70) and particle diffusion (R^2^ = 0.75) also contribute to the process. The higher film diffusion coefficient (d_f_ = 1.04 × 10^−9^ m^2^ s^−1^) compared to particle diffusion (d_p_ = 1.73 × 10^−11^ m^2^ s^−1^) indicates that internal diffusion is comparatively slower once tetracycline molecules reach the hydrochar surface [59].

Overall, the kinetic behavior reflects the combined influence of surface interactions and diffusion processes, with no single mechanism acting as the sole rate-controlling step for tetracycline adsorption onto HC-AW. This interpretation is consistent with SEM observations (Figure 4), which reveal a heterogeneous surface with limited pore development. Considering the relatively large molecular size of tetracycline (14.8 × 9.00 × 7.47 Å) [60], adsorption is likely favored on external surfaces and accessible domains, while diffusion into narrower regions may be restricted.

#### 2.3.3. Adsorption Isotherms

The equilibrium adsorption of tetracycline (TC) onto annatto-derived hydrochar (HC-AW) was evaluated at 294.15, 299.15, and 304.15 K, and the experimental data were fitted using the Freundlich (Equation (6)) and Langmuir (Equation (7)) isotherm models, with parameters summarized in Table 3. These models describe adsorption assuming a distribution of site energies (Freundlich) [61] and uniform adsorption energies (Langmuir) [62].

(6) $$\log q_{e}=\log k_{F}+\frac{1}{n}\log c_{e}$$

where k_F_ is the Freundlich constant associated (L·mg^−1^), c_e_ is the equilibrium adsorbate concentration (mg·L^−1^), and n reflects adsorption intensity and surface heterogeneity.

(7) $$\frac{c_{e}}{q_{e}}=\frac{c_{e}}{q_{m}}+\frac{1}{k_{L}q_{m}}$$

where q_m_ is the maximum adsorption capacity (mg·g^−1^), and k_L_ is the Langmuir constant (L·mg^−1^); adsorption favorability process is evaluated using the dimensionless separation factor r_L_ (Equation (8)).

(8) $$r_{L}=\frac{1}{1+K_{L}c_{o}}$$

The Langmuir model provided an excellent fit (R^2^ = 0.99) at all temperatures, while the Freundlich model also showed good agreement (R^2^ = 0.97–0.98), indicating that both models adequately describe the adsorption behavior. According to the Langmuir model, the maximum adsorption capacity increased from 8.84 mg·g^−1^ at 294.15 K to 14.94 mg·g^−1^ at 304.15 K, suggesting enhanced adsorption at higher temperatures. A slight increase in Langmuir affinity constant (k_L_) was also observed, indicating favorable adsorption under the studied conditions.

The Freundlich parameters further support this behavior, with the heterogeneity factor (1/n) ranging from 0.74 to 0.79, indicating favorable adsorption on a moderately heterogeneous surface. Values of 1/n below unity suggest preferential adsorption at higher-energy sites, with decreasing favorability as surface coverage increases [59].

Although both models describe the data well, the slightly higher correlation of the Langmuir model suggests that adsorption occurs predominantly on a finite number of sites. However, given the concurrent applicability of the Freundlich model, the tetracycline adsorption process likely involves contributions from both homogeneous and heterogeneous surface domains, rather than a single idealized mechanism.

#### 2.3.4. Thermodynamic Analysis of Tetracycline Adsorption onto Annatto-Derived Hydrochar

The thermodynamic parameters governing tetracycline (TC) adsorption onto annatto-derived hydrochar (HC-AW) were determined from equilibrium experiments conducted at different temperatures. The corresponding values of the equilibrium constant (kc), Gibbs free energy change (ΔG°, kJ·mol^−1^), enthalpy change (ΔH°, kJ·mol^−1^), and entropy change (ΔS°, kJ·mol^−1^·K^−1^) are summarized in Table 4, and were calculated using the van’t Hoff equation (Equation (9)) [63].

(9) $$\ln k_{c}=\frac{-{\Delta H}^{0}}{R}\times \frac{1}{T}+\frac{{\Delta S}^{0}}{R}$$

The dimensionless equilibrium constant k_c_ was obtained from the Langmuir constant k_L_ by multiplying it by the molecular weight of the adsorbate (M_w_, g·mol^−1^) and applying a factor of 1000, which corresponds to the number of moles of pure water per liter, as described in Equation (10) [64]. Here, R is the universal gas constant (8.314 J·mol^−1^·K^−1^), and T is the absolute temperature (K).

(10) $$k_{c}=k_{L}\times M_{w}\times 1000$$

The equilibrium constant values (expressed as ln kc) remain relatively consistent across the studied temperature range, with values of 8.74, 8.76, and 8.77 at 294.15, 299.15, and 304.15 K, respectively. This stability suggests that the adsorption equilibrium is not significantly affected within the investigated temperature interval. The linear correlation obtained in the van’t Hoff analysis (R^2^ = 1.00) indicates a good fit of the experimental data within the studied range.

The calculated Gibbs free energy changes (ΔG°) are −21.37, −21.78, and −22.19 kJ mol^−1^ for the three investigated temperatures, respectively. The negative values of ΔG° indicate that the adsorption process is thermodynamically favorable under the studied conditions. Furthermore, the slight increase in the magnitude of ΔG° with increasing temperature suggests that this favorability is maintained across the examined temperature range [59].

The enthalpy change (ΔH°) obtained from the van’t Hoff relationship is 2.59 kJ mol^−1^, indicating a small positive enthalpy change associated with the adsorption process. The relatively low magnitude of ΔH° suggests that the energy change accompanying adsorption is limited, which may be consistent with weak interaction forces. This value also indicates that the process is slightly endothermic, although its magnitude does not allow a definitive mechanistic assignment. The entropy change (ΔS°) calculated for the system is 0.08 kJ·mol^−1^·K^−1^, indicating a positive variation in entropy during the adsorption process. This positive value suggests an increase in disorder at the solid–solution interface, which may be associated with changes in solvation and adsorbate–surface interactions [59]. Overall, these thermodynamic parameters provide insight into the energetic aspects of the adsorption process.

### 2.4. Mechanistic Considerations and Comparative Assessment

The adsorption mechanism of tetracycline (TC) onto annatto-derived hydrochar (HC-AW) can be interpreted by integrating physicochemical characterization (FTIR, SEM) with adsorption results (e.g., kinetics, equilibrium isotherms, and pH-dependent adsorption behavior), providing a consistent framework to rationalize the observed behavior.

The pH-dependent experiments indicate that adsorption is favored under near-neutral conditions (pH ≈ 7), where tetracycline predominantly exists in its zwitterionic form (pKa_1_ ≈ 3.3, pKa_2_ ≈ 7.7, pKa_3_ ≈ 9.7) [50], and the hydrochar surface approaches charge neutrality (pH_PZC_ of HC-AW is 6.3 ± 0.5). Under these conditions, electrostatic repulsion is minimized, facilitating the establishment of intermolecular interactions at the adsorbent surface.

**Figure 7:**
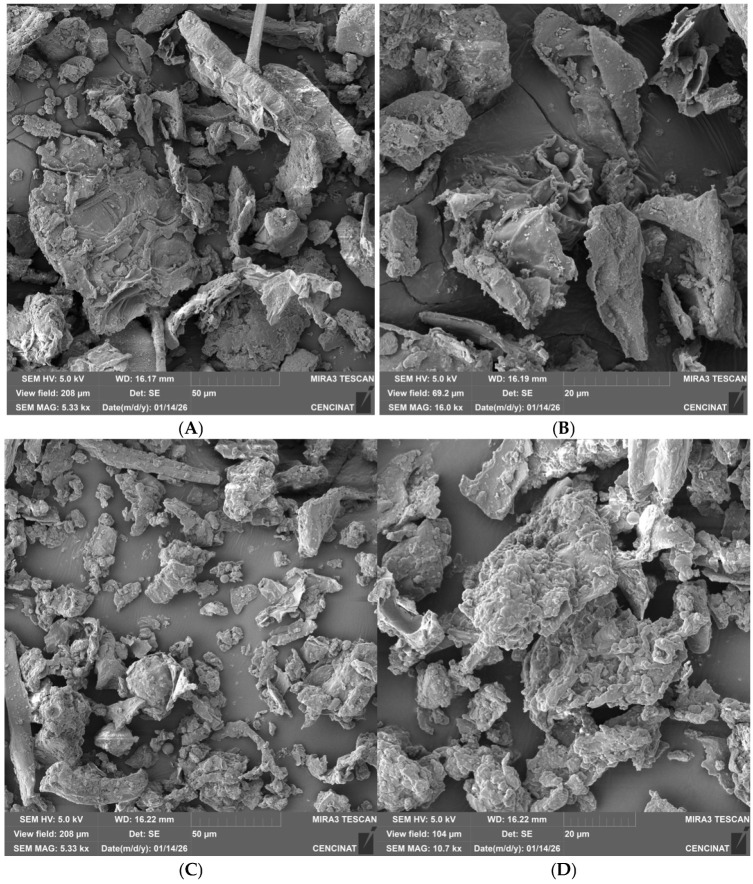
SEM micrographs of annatto-derived hydrochar (HC-AW) before and after tetracycline adsorption at different magnifications: (**A**,**B**) pristine hydrochar surface and (**C**,**D**) hydrochar surface after tetracycline adsorption.

SEM observations after adsorption (Figure 7) show partial coverage by irregular deposits and the masking of accessible cavities, indicating accumulation of tetracycline on the hydrochar surface. Considering the relatively large molecular size of tetracycline (14.8 × 9.00 × 7.47 Å) [60] and the limited pore development of HC-AW, adsorption is likely favored on external and accessible surface domains, while diffusion into narrower regions may be restricted.

Kinetic analysis further supports this interpretation. Although the pseudo-second-order model provides an excellent fit (R^2^ = 1.00), this does not imply a single controlling mechanism. Instead, the combined evidence from diffusion models indicates that both surface interactions and mass-transfer processes contribute to adsorption, with no single step acting as the sole rate-limiting factor.

Equilibrium results are well described by the Langmuir model, while the concurrent applicability of the Freundlich model suggests contributions from both homogeneous and heterogeneous surface domains. The increase in adsorption capacity with temperature further indicates that tetracycline–hydrochar surface interactions remain favorable within the studied range.

**Figure 8:**
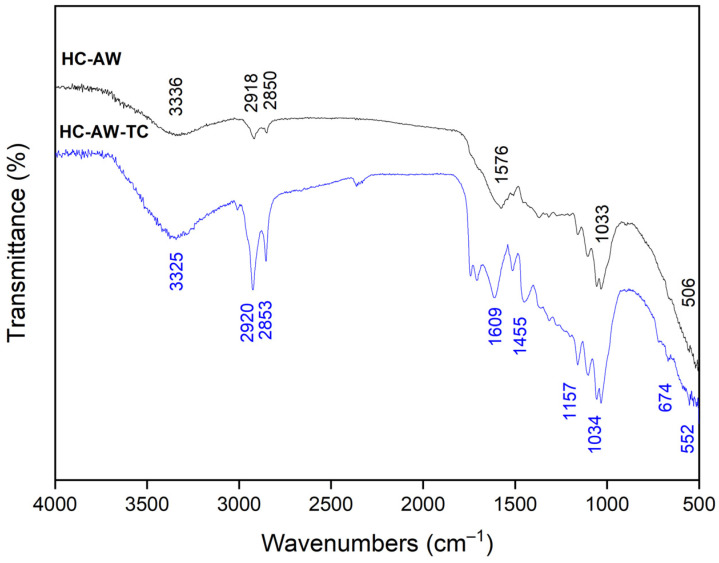
FTIR spectra of annatto-derived hydrochar (HC-AW) before and after tetracycline adsorption (HC-AW and HC-AW–TC).

The FTIR spectra provide spectroscopic evidence of the interaction between tetracycline (TC) and the surface functionalities of HC-AW (Figure 8). After adsorption, systematic changes are observed in key spectral regions, indicating the involvement of oxygen-containing functional groups in the adsorption process.

The broad O–H band (3350–3330 cm^−1^) shows reduced intensity and slight broadening, suggesting participation of hydroxyl groups in hydrogen bonding interactions [65]. Similarly, modifications in the aromatic C=C region (1610–1600 cm^−1^) indicate interactions between the conjugated carbonyl structures of tetracycline and the hydrochar matrix, consistent with π–π interactions [66]. Changes in the C–O stretching region (1040–1020 cm^−1^). further support the involvement of oxygenated functionalities in the adsorbate-surface interactions; likely associated with surface complexation or hydrogen bonding [67]. Additional variations in the carbonyl region (1740–1700 cm^−1^) suggest a possible contribution of oxygenated groups acting as interaction sites, although these changes are less pronounced [68].

The spectroscopic features are consistent with the speciation of tetracycline at near-neutral pH, where the molecule exhibits both donor and acceptor functionalities, facilitating multiple interaction pathways. Overall, the FTIR results support that adsorption involves the combined contribution of surface interactions associated with oxygen-containing functional groups (e.g., hydroxyl, carbonyl, aromatic, and C–O), in agreement with the mechanistic interpretation derived from pH, kinetic and morphological analyses.

To contextualize the adsorption performance of the annatto-derived hydrochar (HC-AW), Table 5 compares its capacity with that of biomass-derived carbonaceous adsorbents reported in the literature. While tetracycline removal has been widely investigated using engineered materials and conventional carbon adsorbents, studies focusing on hydrochars derived from underutilized agroindustrial residues remain limited. In this regard, annatto (*Bixa orellana*) processing waste represents a scarcely explored precursor, despite its potential availability. The HC-AW material exhibits adsorption performance within the range reported for hydrochar-based systems, indicating that effective tetracycline removal can be achieved without the need for high-temperature treatments or extensive material modification. From a comparative perspective, differences in adsorption behavior among materials can be associated with variations in synthesis route, surface functionality, and structural accessibility. In particular, hydrothermal carbonization produces materials with higher oxygen-containing functionality and less developed porosity compared to pyrolytic biochars, which may influence adsorption toward large and multifunctional molecules such as tetracycline [9]. Overall, the results position annatto-derived hydrochar as a viable adsorbent within the broader context of biomass-based materials, while highlighting the relevance of precursor selection and synthesis conditions in defining adsorption performance.

**Table 5:** Comparison of tetracycline adsorption capacities reported for hydrochar and biochar materials derived from biomass residues.

Adsorbent	Precursor/Treatment	Type	Adsorption Capacity (mg·g^−1^)	Experimental Conditions	Reference
Annatto-derived hydrochar (HC-AW)	*Bixa orellana* residue; HTC 200 °C	Hydrochar	14.94	pH 7, 30 °C	This work
Orange-peel hydrochar	Orange peel; HTC 190 °C	Hydrochar	65.24	pH 7, 25 °C	[69]
HNO_3_-modified orange-peel hydrochar	Orange peel hydrochar + HNO_3_ oxidation	Modified hydrochar	126.54		
Sugarcane-bagasse hydrochar	Sugarcane bagasse; HTC 2 M HCl, 180 °C	Hydrochar without acid-assisted	33.6	pH 5, 26 °C	[70]
	Sugarcane bagasse; HCl-assisted HTC	Modified hydrochar optimized hydrochar	68.2		
Engineered paddy-husk hydrochar	Paddy husk; HTC 220 °C 20% H3PO4	Hydrochar modified	47	pH 3, 27 °C	[65]
Woody biomass	Woody biomass HTC-C	Hydrochar	56	pH 7, 25 °C	[71]
	Woody biomass HTC-C with the addition of K2FeO	Modified Hydrochar	119.7		
Rice-straw biochar	Rice straw; pyrolysis 700 °C	Biochar	14	pH 5.5; 35 °C	[72]
Swine manure-derived biochar	Swine manure-derived biochar	Biochar	67.57	Non reported	[73]
	Swine manure-derived biochar (SBC) modified with KMnO_4_	Biochar	95.81		
Corn-straw biochar (CSB)	Corn straw; pyrolysis 500 °C	Biochar	8.22	pH 6, 25 °C	[74]
Ca(OH)_2_-modified corn-straw biochar (Ca-CSB)	Corn straw biochar + Ca(OH)_2_ modification	Modified biochar	93.46		

From a process–property perspective, the adsorption behavior of HC-AW can be related to the relatively mild hydrothermal carbonization conditions (200 °C, 3 h), which promote partial dehydration and structural reorganization while preserving oxygen-containing surface functionalities. As reported in the literature, hydrochars produced within the 180–220 °C range typically retain polar functional groups favorable for adsorption, whereas more severe treatments enhance aromaticity and porosity at the expense of surface polarity [75,76]. In this context, the adsorption response of HC-AW is consistent with a material in which oxygenated functionalities remain available for pH-dependent interactions, while limited pore development constrains adsorption capacity.

From a comparative perspective, the adsorption capacity of HC-AW (14.94 mg g^−1^) falls within the lower–moderate range reported for pristine hydrochars and unmodified biochars, which commonly exhibit values below 40 mg g^−1^ due to limited surface area and pore accessibility. In contrast, chemically modified or activated materials (e.g., heteroatom or metal-oxide doping) often exceed 100 mg·g^−1^ as a result of enhanced porosity and additional functionalization. Although HC-AW does not reach the performance of these engineered systems, its adsorption behavior is consistent with low-temperature hydrochars and remains competitive considering its mild synthesis conditions, absence of chemical activation, and sustainable origin. Notably, the effective adsorption under near-neutral conditions further supports its practical applicability in real water treatment scenarios. However, the moderate adsorption capacity suggests that further optimization of surface area, pore accessibility, or surface chemistry may be required for high-load applications.

### 2.5. Limitations and Future Perspectives

To provide a balanced interpretation of the results, the main limitations of this study and directions for future research are outlined, with emphasis on material variability, process conditions, and practical applicability.

A key limitation is the inherent variability of annatto agroindustrial residue, which may depend on raw material origin, processing conditions, and pigment extraction efficiency. Such variability can influence the physicochemical properties of the resulting hydrochar and, consequently, its adsorption performance. In addition, the potential presence of residual lipophilic compounds derived from industrial extraction processes cannot be excluded and may affect hydrothermal carbonization pathways and material reproducibility. The pre-treatment sequence applied in this study (drying, alkaline washing, and re-drying) was implemented to reduce heterogeneity and remove impurities; however, it may increase water and energy consumption. Therefore, future work should focus on optimizing pre-treatment strategies to improve process sustainability while ensuring reproducible material properties.

The hydrothermal synthesis conditions evaluated here represent a specific set of parameters optimized for tetracycline removal. However, variations in temperature, residence time, and solid–liquid ratio are known to significantly affect hydrochar structure and surface chemistry. As adsorption behavior depends on the interplay between adsorbent properties and adsorbate characteristics, further studies are required to systematically explore process–structure–property relationships and to identify optimal conditions for different classes of contaminants.

From an application perspective, adsorption experiments were conducted under controlled conditions using single-solute systems. The performance of HC-AW in more complex water matrices, where competing ions and dissolved organic matter may influence adsorption behavior, remains to be assessed. Moreover, regeneration and reusability were not evaluated and should be addressed in future studies to determine the long-term applicability of the material.

Despite these limitations, HC-AW exhibits several relevant advantages. Notably, its maximum adsorption performance occurs under near-neutral pH conditions, which are representative of most natural and treated water systems. This feature constitutes a practical advantage over many high-performance adsorbents that require acidic or alkaline conditions to achieve optimal uptake, often increasing operational costs and limiting real-world applicability. In contrast, the ability of HC-AW to operate effectively without pH adjustment enhances its practical feasibility for sustainable water treatment applications.

Overall, annatto-derived hydrochar represents a promising low-cost adsorbent produced under mild processing conditions from an underutilized agroindustrial residue. While its adsorption capacity remains moderate compared to engineered materials, its operational simplicity, favorable performance under environmentally relevant conditions, and sustainable origin highlight its potential for practical implementation. Future research should focus on improving surface area, accessibility, and functionality through targeted modifications, as well as validating performance under real environmental conditions.

## 3. Materials and Methods

### 3.1. Collection and Pretreatment of Annatto Agroindustrial Residue

Annatto (*Bixa orellana*) agroindustrial residue was obtained from a local food-processing facility after pigment extraction. The collected material was transported to the laboratory and initially dried to remove residual moisture. This step was also intended to facilitate handling and homogenize the moisture content, as the residue was originally stored under open-air conditions. Drying was performed in a forced-air oven at 80 °C for 24 h until constant weight. The dried residue was subsequently washed with 0.1 M NaOH solution at 40 °C to remove soluble impurities and residual organic compounds. Considering that pigment extraction in food-processing applications commonly involves edible oils, this treatment was also intended to partially remove residual lipophilic compounds that could interfere with subsequent processing. Additionally, this step contributed to reducing potential microbial contamination associated with storage conditions. After alkaline washing, the material was thoroughly rinsed with distilled water until a neutral pH was reached. The pretreated residue was then dried again at 80 °C for 24 h, ground using a mortar, and sieved through a 35-mesh screen to obtain a homogeneous particle size suitable for hydrothermal processing. This pre-treatment sequence was designed to ensure consistent feedstock properties prior to hydrothermal carbonization in an aqueous medium. The second drying step ensured consistent experimental conditions by homogenizing multiple residue batches into a single feedstock with comparable properties.

### 3.2. Hydrothermal Carbonization of Annatto Waste

Hydrothermal carbonization (HTC) was performed using a laboratory-scale stainless-steel autoclave reactor with a total volume of 100 mL. The pretreated annatto residue (5 g) was mixed with distilled water (25 mL) at a solid-to-liquid ratio of 1:5 (*w*/*v*) to provide the reaction medium for hydrothermal conversion. The selected operating conditions (200 °C, 3 h, and 1:5 solid-to-liquid ratio) were defined based on preliminary laboratory-scale experiments to ensure stable hydrochar formation; adsorption performance at different HTC temperatures was evaluated but is not reported, as this study focuses on optimized conditions. In the absence of prior studies on annatto-derived residues, the process parameters were experimentally defined. These parameters are consistent with commonly reported HTC ranges for lignocellulosic biomass (180–240 °C, ~3 h), where temperature is the primary factor controlling process severity [32].

The reactor (Baoshishan, China) was hermetically sealed, and no external gas was introduced during the process; therefore, the system operated under autogenous pressure generated by water at the reaction temperature (200 °C). The pressure was not directly measured during the experiments. The sealed reactor was placed in a convection oven (Memmert, Schwabach, Germany) at 200 °C for 3 h. After completion of the reaction, the system was allowed to cool naturally to approximately 50 °C before opening. The resulting solid product was separated from the liquid phase by vacuum filtration and extensively washed with distilled water (5–6 L) until a neutral pH was reached. The recovered hydrochar was dried at 105 °C for 24 h and subsequently sieved through a 200-mesh screen to obtain a uniform powder. The hydrochar yield was calculated as the ratio between the mass of dry hydrochar obtained and the initial dry mass of annatto residue.

### 3.3. Physicochemical Characterization of Hydrochar

The physicochemical properties of the raw material (AW) and the synthesized hydrochar (HC-AW) were evaluated by X-ray fluorescence (XRF) using a handheld XRF analyzer (Bruker Titan 800, Bruker, Billerica, MA, USA), allowing the quantification of major and minor inorganic oxides present in the material. The crystalline structure of the hydrochar was analyzed by X-ray diffraction (XRD) using a D8 Advance A25 diffractometer (Bruker, Karlsruhe, Germany) equipped with Cu Kα radiation (λ = 1.5406 Å) operating at 40 kV and 40 mA. Diffraction patterns were recorded over a 2θ range of 5–80°. Surface functional groups were characterized by Fourier transform infrared spectroscopy (FTIR) using a Nicolet iS10 spectrometer (Thermo Scientific, Waltham, MA, USA). Spectra were collected in the 4000–400 cm^−1^ wavenumber range using the KBr pellet method, with 32 scans recorded at a resolution of 4 cm^−1^. The surface morphology and microstructural features of the hydrochar were examined by scanning electron microscopy (SEM) using a field emission scanning electron microscope (Tescan Mira 3, Brno, Czech Republic) equipped with an energy-dispersive X-ray spectroscopy (EDS) detector. SEM analysis provided information on particle morphology and surface texture, while EDS enabled semi-quantitative elemental analysis at the surface level; however, it may not fully represent the bulk composition of the hydrochar. Additionally, the point of zero charge (pH_PZC_) was determined using the salt addition method. In this procedure, hydrochar suspensions (0.1 g) were equilibrated in deionized water and 0 NaCl solutions at concentrations of 0.01 and 0.05 M. The initial pH of each suspension was adjusted between 2 and 11. After 24 h of equilibration, the final pH was recorded, and the pHPZC was determined from the intersection point between the initial and final pH values.

### 3.4. Batch Adsorption Experiments

Batch adsorption experiments were carried out to evaluate the performance of annatto-derived hydrochar (HC-AW) for tetracycline removal from aqueous solution at room temperature (25 ± 2 °C). In each experiment, 0.1 g of HC-AW was equilibrated with 25 mL of tetracycline solution at the established initial concentration under constant agitation. Blank experiments were performed in the absence of adsorbent to verify that no significant tetracycline loss occurred due to photolysis or adsorption onto the container walls. After the adsorption period, the suspensions were filtered and the residual tetracycline concentration in the supernatant was determined by UV–Vis spectrophotometry at 357 nm, corresponding to the maximum absorbance wavelength of tetracycline in aqueous solution.

The influence of pH on tetracycline adsorption was evaluated using TC solutions in the range pH 3 to 11, adjusted with diluted HCl or NaOH solutions before the addition of the adsorbent. The equilibrium adsorption capacity was calculated according to Equation (11).

(11) $$q_{e}=\frac{v\;x\;(c_{0}-c_{e})}{w}$$

where v is the solution volume (L), c_0_ and c_e_ are the initial and equilibrium TC concentrations (mg·L^−1^), and w is the adsorbent mass (g).

Adsorption kinetics were investigated at pH 7 by collecting samples at predetermined contact times ranging from a few minutes up to 24 h. The adsorption capacity at time t was calculated according to Equation (12):

(12) $$q_{t}=\frac{v\;x\;(c_{0}\;-{\;c}_{t})}{w}$$

where c_t_ is the tetracycline concentration at time t (mg·L^−1^ TC).

Equilibrium adsorption isotherms were determined by equilibrating HC-AW with tetracycline solutions of different initial concentrations ranging from 5 to 200 mg·L^−1^ at pH 7. The equilibrium data were subsequently used to evaluate the maximum adsorption capacity of the hydrochar using the Langmuir model.

Thermodynamic experiments were performed at 294.15, 299.15, and 304.15 K, maintaining identical experimental conditions to determine the thermodynamic parameters governing the adsorption process. All experiments were conducted in triplicate, and the values reported in figures and tables correspond to the mean values with their respective standard deviations.

## 4. Conclusions

This study demonstrates the feasibility of converting annatto (*Bixa orellana*) agroindustrial residue into a functional hydrochar adsorbent for tetracycline removal from aqueous solutions through hydrothermal carbonization. The process yielded a carbonaceous material with a solid recovery of 44%. The resulting hydrochar exhibited a predominantly amorphous carbon structure with retained oxygen-containing functionalities, characteristic of materials produced under subcritical conditions, confirming effective biomass conversion.

Batch adsorption experiments revealed that tetracycline uptake is strongly influenced by solution pH, with maximum adsorption occurring under near-neutral conditions (pH ≈ 7), where tetracycline predominantly exists in its zwitterionic form and electrostatic repulsion with the hydrochar surface is minimized. This behavior represents a practical advantage, as it enables operation under environmentally relevant conditions without the need for pH adjustment.

Kinetic analysis showed that the adsorption process is well described by the pseudo-second-order model; however, no definitive mechanistic assignment can be established from kinetic modeling alone, as multiple interaction regimes may coexist, involving both surface interactions and mass-transfer processes. Diffusion analysis indicated that both film diffusion and intraparticle diffusion contribute to the adsorption process, without either acting as the sole rate-controlling step. Equilibrium studies demonstrated that the Langmuir model adequately describes the adsorption behavior, with a maximum adsorption capacity of 14.94 mg·g^−1^ at 30 °C, suggesting predominantly monolayer adsorption on energetically similar sites while not excluding surface heterogeneity. Thermodynamic analysis confirmed that the adsorption process is spontaneous and slightly endothermic within the investigated temperature range. Spectroscopic and morphological analyses provided complementary evidence that adsorption involves multiple interaction mechanisms, including hydrogen bonding, π–π interactions, and surface complexation. Additionally, morphological features and the relatively large molecular size of tetracycline suggest that adsorption occurs predominantly on external and accessible surface domains rather than within well-defined porous networks. Overall, this work expands the range of biomass precursors investigated for hydrochar production and demonstrates that annatto processing waste can be transformed into a sustainable and low-cost adsorbent. Furthermore, it provides integrated mechanistic insight into the relationships between structure, surface chemistry, and adsorption behavior, establishing a basis for future optimization of hydrochar materials and their application in antibiotic removal from aqueous systems.

## Figures and Tables

**Table 1:** Chemical composition of annatto agroindustrial waste (AW) and annatto-derived hydrochar (HC-AW) obtained by X-ray fluorescence analysis.

Chemical Composition	Annatto Waste (%)	Annatto-Derived Hydrochar (%)
MgO	−	2.72 ± 0.51
Al_2_O_3_	0.15 ± 0.04	−
SiO_2_	0.39 ± 0.02	0.55 ± 0.03
P_2_O_5_	0.18 ± 0.04	0.06 ± 0.02
S	0.23 ± 0.04	0.21 ± 0.01
K_2_O	1.65 ± 0.05	0.80 ± 0.02
CaO	0.72 ± 0.02	0.80 ± 0.04
MnO	0.04 ± 0.00	0.04 ± 0.00
Fe_2_O_3_	0.03 ± 0.00	0.05 ± 0.00
Zn	0.01 ± 0.00	−
Cr	0.004 ± 0.000	0.004 ± 0.000
Sr	0.004 ± 0.000	0.004 ± 0.000
Pb	0.011 ± 0.000	0.011 ± 0.000

The reported values correspond to relative oxide percentages determined by XRF within the detectable inorganic fraction and do not represent the total mass composition of the materials.

**Table 2:** Kinetic parameters for tetracycline adsorption onto annatto-derived hydrochar (HC-AW).

Model	Parameter	Value
Pseudo-first order	q_e_ (mg∙g^−1^)	0.55
	k_1_ (h^−1^)	0.27
	R^2^	0.70
Pseudo-second order	q_e_ (mg∙g^−1^)	1.68
	k_2_ (g∙mg^−1^∙h^−1^)	6.90
	R^2^	1.00
Intraparticular diffusion	k_t1_ (mg∙g^−1^∙h^−1/2^)	6.79
	R^2^	0.97
	k_t2_ (mg∙g^−1^∙h^−1/2^)	0.47
	R^2^	0.97
	k_t3_ (mg∙g^−1^∙h^−1/2^)	0.03
	R^2^	0.99
Film diffusion	d_f_ (m^2^∙h^−1^)	1.04 × 10^−9^
	R^2^	0.70
Particle diffusion	d_p_ (m^2^∙h^−1^)	1.73 × 10^−11^
	R^2^	0.75

**Table 3:** Isotherm parameters for tetracycline adsorption onto annatto-derived hydrochar at different temperatures (HC-AW).

Isotherm Model		Kinetic Parameters		
		T= 294.15 °C	T= 299.15 °C	T= 304.15 °C
Langmuir	q_m_ (mg∙g^−1^)	8.84	11.76	14.94
	k_L_ (L∙g^−1^)	1.41 × 10^−2^	1.43 × 10^−2^	1.46 × 10^−2^
	R^2^	0.99	0.99	0.99
Freundlich	k_F_ (mg∙g^−1^)	0.28	0.23	0.25
	1/n	0.79	0.74	0.79
	R^2^	0.98	0.98	0.97

**Table 4:** Thermodynamic parameters for tetracycline adsorption onto annatto-derived hydrochar (HC-AW).

Temperature	ln k_c_	R^2^	ΔG^0^	ΔS^0^	ΔH^0^
(K)			(kJ·mol^−1^)	(kJ·mol^−1^·K^−1^)	(kJ·mol^−1^)
294.15	8.74	1.00	−21.37	0.08	2.59
299.15	8.76		−21.78		
304.15	8.77		−22.19		

## Data Availability

Data are contained within the article.

## Supplementary Materials

The following supporting information can be downloaded at: https://www.mdpi.com/article/10.3390/molecules31071224/s1, Figure S1. Energy-dispersive X-ray spectroscopy (EDS) spectrum of annatto agroindustrial waste (AW), confirming carbon and oxygen as the dominant elements together with minor inorganic constituents; Figure S2. Energy-dispersive X-ray spectroscopy (EDS) spectrum of annatto-derived hydrochar (HC-AW), evidencing the predominance of carbon in the hydrochar matrix and supporting the compositional trends observed by XRF.
