# Supplementary material for: Hydrothermal Conversion of Annatto Seed Waste (Bixa orellana) into Functional Hydrochar: Synthesis, Characterization, and Adsorption Mechanism of Tetracycline
Source: Molecules. 2026 Apr 7;31(7):1224. doi: 10.3390/molecules31071224 (PMC13074624; doi:10.3390/molecules31071224)
Supplement: Supplementary file 1 [file molecules-31-01224-s001.zip › molecules-4228641-supplementary.pdf]

Supplementary Materials

Hydrothermal Conversion of Annatto Seed Waste (*Bixa orellana*) into Functional Hydrochar: Synthesis, Characterization, and Adsorption Mechanism of Tetracycline

Diana Guaya [1*], Linda Jadán [2] and José Luis Cortina [3,4]

[1] Department of Chemistry, Universidad Técnica Particular de Loja, Loja 110107. Ecuador
[2] Escuela de Ingeniería Química, Universidad Técnica Particular de Loja. Loja 110107. Ecuador; lijadan@utpl.edu.ec
[3] Department of Chemical Engineering, Polytechnic University of Catalonia–BarcelonaTech (UPC), 08019 Barcelona, Spain; jose.luis.cortina@upc.edu
[4] Barcelona Research Center in Multiscale Science and Engineering (CCEM), Universitat Politècnica de Catalunya-BarcelonaTech (UPC), Av. Eduard Maristany, 16, Barcelona 08019, Spain.

* Correspondence: deguaya@utpl.edu.ec

Figure S1. Energy-dispersive X-ray spectroscopy (EDS) spectrum of annatto agroindustrial waste (AW), confirming carbon and oxygen as the dominant elements together with minor inorganic constituents.

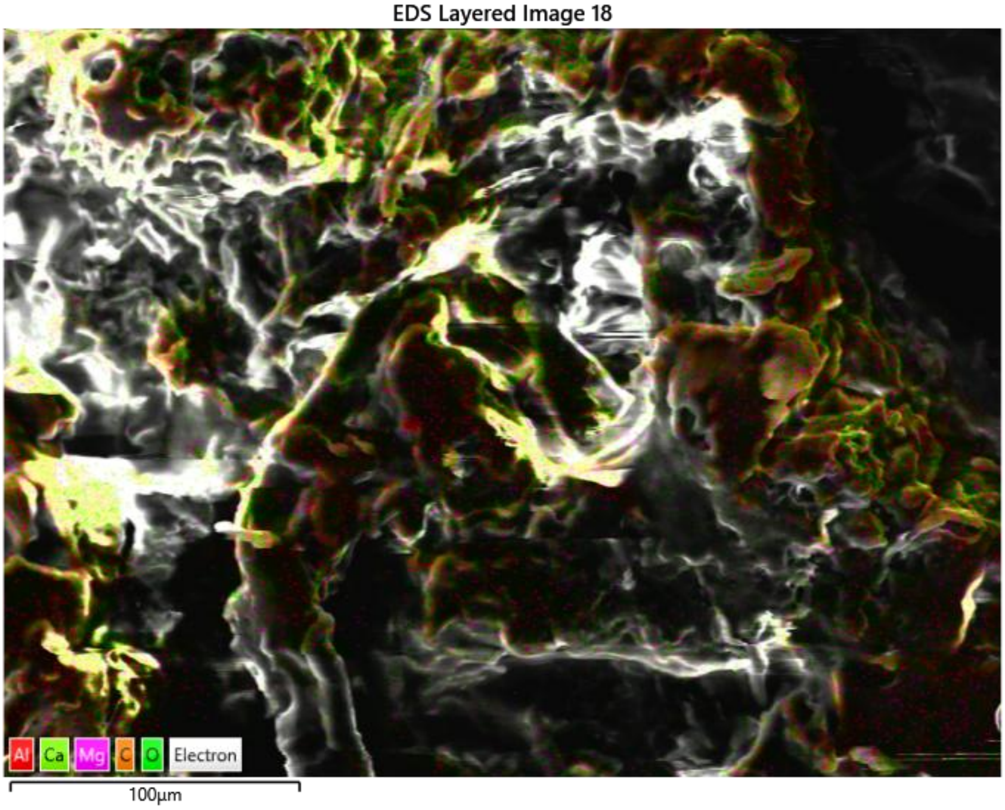

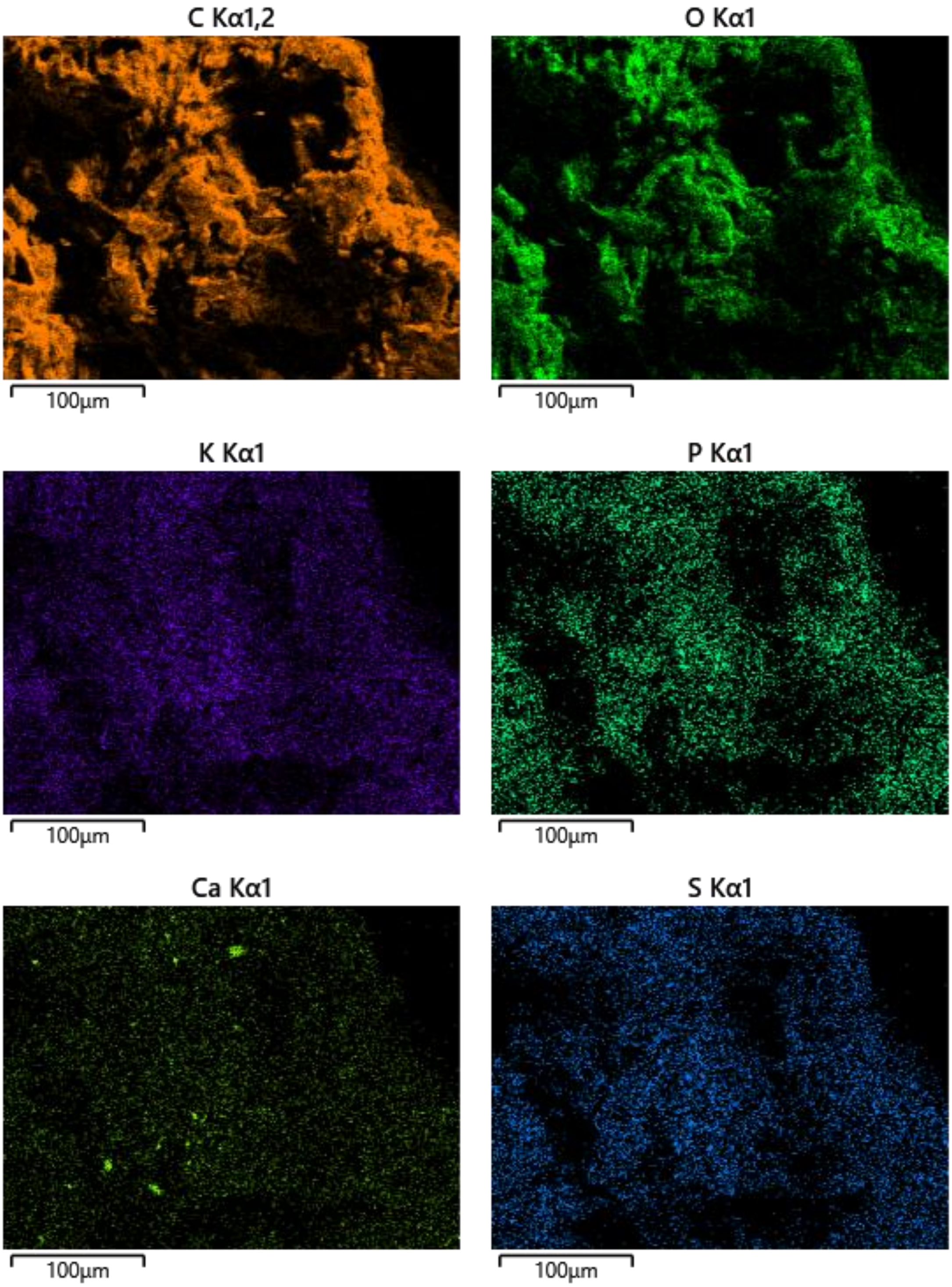
C Kα1,2
O Kα1
100µm
100µm
K Kα1
P Kα1
100µm
100µm
Ca Kα1
S Kα1
100µm
100µm

Cl Kα1

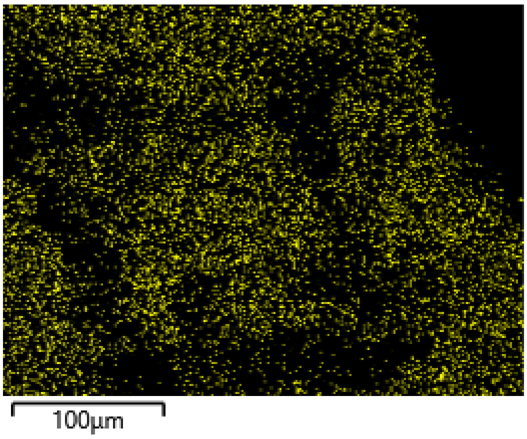

Mg Kα1,2

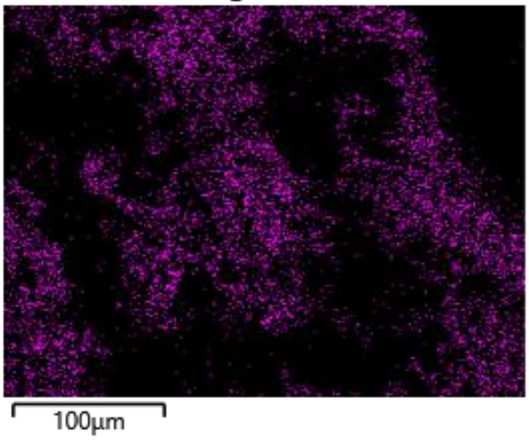

Al Kα1

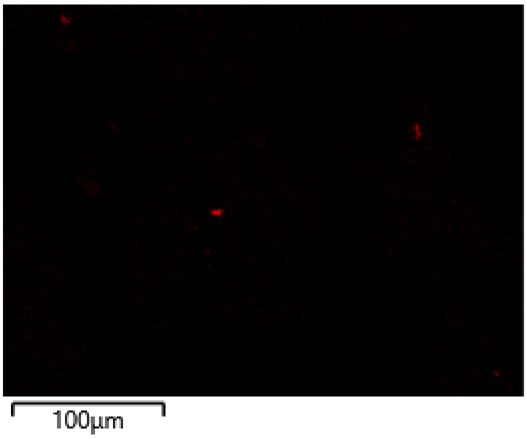

Figure S2. Energy-dispersive X-ray spectroscopy (EDS) spectrum of annatto-derived hydrochar (HC-AW), evidencing the predominance of carbon in the hydrochar matrix and supporting the compositional trends observed by XRF.

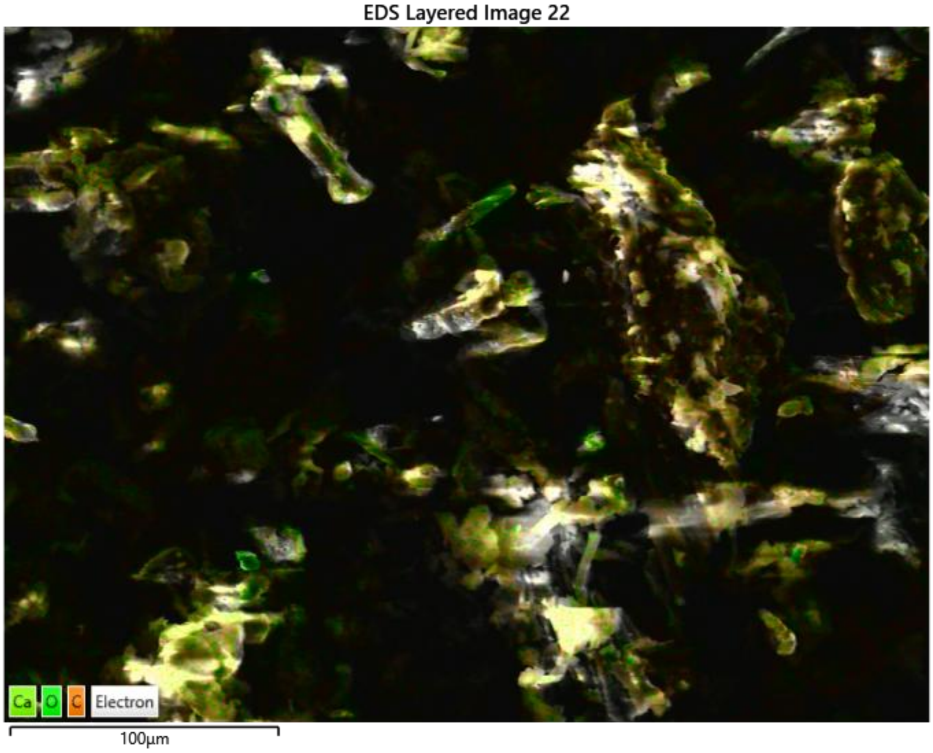

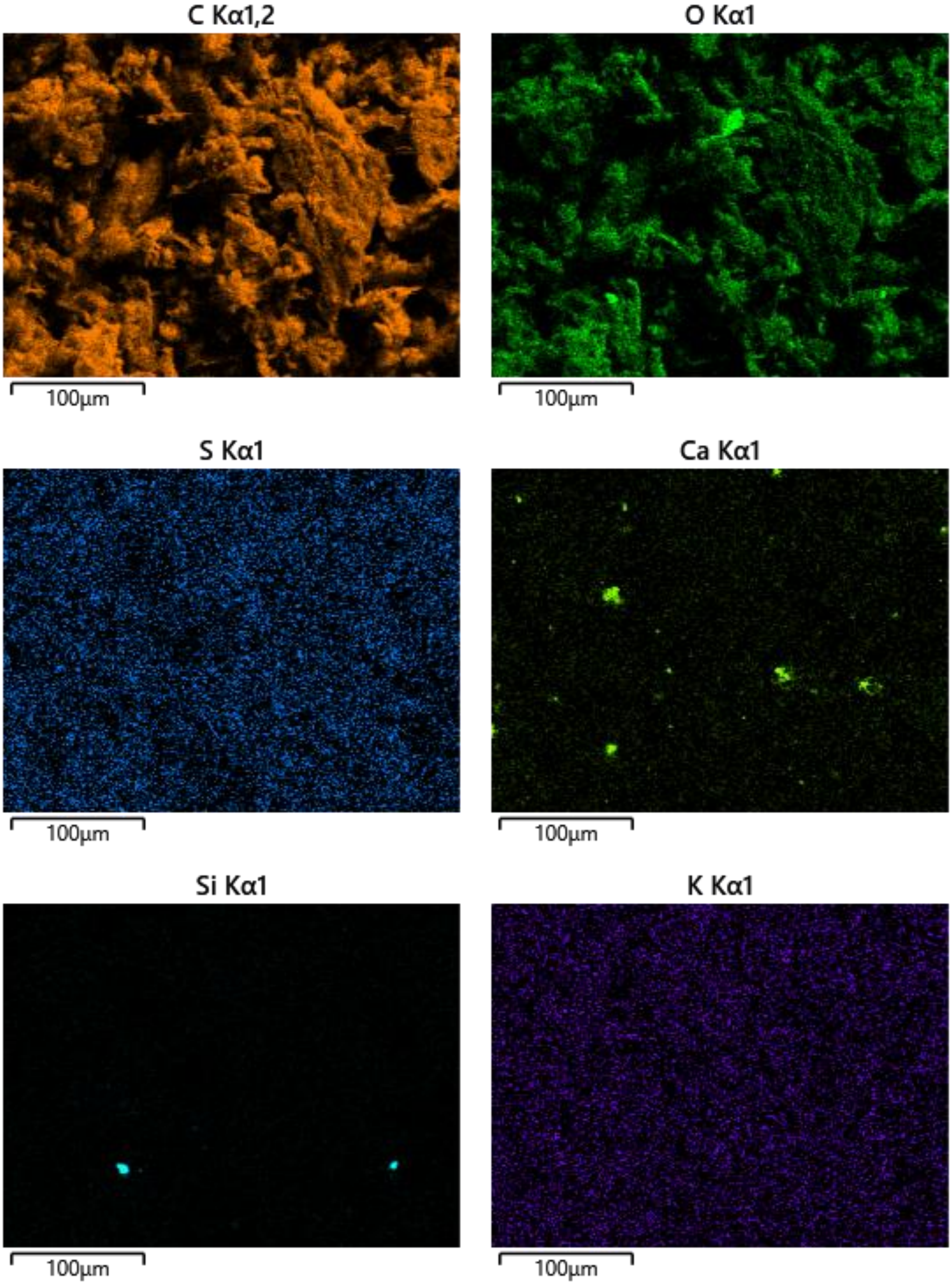
C Kα1,2
100µm
O Kα1
100µm
S Kα1
100µm
Ca Kα1
100µm
Si Kα1
100µm
K Kα1
100µm
